# Assessment of manometric results following posterior pericervical repair or level I to III surgical procedures

**DOI:** 10.25122/jml-2022-0357

**Published:** 2023-12

**Authors:** Maryam Deldar Pesikhani, Reihane Sadat Hosseini, Sanam Ghanbarpour, Sanaz Ghashghaee, Parivash Jelodarian, Maryam Kazemi, Tahereh Eftekhar, Zinat Ghanbari

**Affiliations:** 1 Department of Obstetrics and Gynecology, Faculty of Medicine, Tehran University of Medical Sciences, Tehran, Iran; 2 Department of Obstetrics and Gynecology, Fertility Infertility and Perinatology Research Center, School of Medicine, Ahvaz Jundishapur University of Medical Sciences, Ahvaz, Iran

**Keywords:** defecation, manometry, pelvic organ prolapse (POP), uterine prolapse

## Abstract

Constipation and obstructive bowel disorders are the most common symptoms of prolapse and posterior defects. Prolapse and obstructive defecation disorders are treated using various surgical techniques to repair posterior defects. This study aimed to evaluate the manometry results of patients before and after reconstructive surgery of the posterior compartment. This retrospective cohort study included 40 women with defecation disorders referred to the Imam Khomeini Hospital Complex, an academic center affiliated with Tehran University of Medical Sciences, Tehran, Iran, from 2020 to 2021. Data were collected through medical records and a checklist developed by the researcher before and after surgery. All analyses were performed using SPSS software (version 26), with significance at p<0.05. Forty women with a mean age of 49.47±9.66 years participated in this study. The manometry results showed significant differences in patients before and after surgery in parameters such as maximum resting pressure, push test, constipation, straining during defecation, finger support necessity, sensation of incomplete defecation, dyspareunia, and husband’s sexual satisfaction (p<0.001). In addition, all patients had a grade 2 or higher posterior compartment prolapse, which improved in all cases after surgery (p<0.0001). Patients’ symptoms significantly improved during the 12-month follow-up after DeLancey level 3 to 1 surgery. This type of surgery proved to be an effective surgical intervention without significant complications in the short-term follow-up.

## INTRODUCTION

Pelvic organ prolapse (POP) refers to the descent of one or more portions of the vagina and uterus, including the anterior wall, posterior wall, uterus (cervix), or apex of the vagina (cuff scar after hysterectomy) [1]. The prevalence of POP is significantly lower based on the symptoms reported (3%–6%) than that determined by examination (41%–50%) [2]. This discrepancy may be because many women with POP tend to be asymptomatic. Therefore, although women aged between 45 and 85 with objective POP on examination account for 40% of the general population, only 12% of POP cases are symptomatic [3]. As reported in different studies, POP prevalence ranges from 3% to 50% [2, 4, 5]. According to reports, 53% of Iranian women suffer from POP [6].

Weakening and aging of the pelvic floor muscles can lead to complications, such as urinary incontinence, cystocele, rectocele, and enterocele. Rectocele, called prolapse of the posterior wall of the vagina or DeLancey level 2 defect, is a protrusion of the rectovaginal wall [7] and can cause constipation and incomplete stool evacuation. Recent studies have suggested that obstructed defecation is significantly correlated with posterior pelvic anatomical defects, including true rectocele, enteric hernia, and intussusception [8]. Based on a recent study, only 39% of women with POP have true rectoceles [9].

Various surgical methods have been developed to repair rectoceles and improve secondary prolapse and obstructive defecation disorder symptoms. Surgery usually improves obstructive disorders by correcting anatomical defects. Several surgical techniques are available for rectocele repair, including posterior colporrhaphy, rectocele-specific defect repair, and sacrocolpoperineopexy [10]. For more than 100 years, gynecological surgeons have used posterior colporrhaphy to repair rectoceles. As part of traditional posterior colporrhaphy, the rectovaginal septum is usually removed, and perineorrhaphy is performed, narrowing the vaginal caliber [11]. According to a review of 46 studies on different types of surgery, transanal repair is associated with fecal incontinence. Both transanal and transvaginal repairs improve prolapse symptoms and defecatory disorders, with transvaginal repair demonstrating superior anatomical outcomes. In some studies, symptoms related to defecation disorders worsened despite the improvement in prolapse symptoms following sacrocolpoperineopexy repair [12]. Although anatomical treatment has been widely used, little data are available on the success of such treatments, as well as the improvement of sexual symptoms. Therefore, it is important to examine and evaluate sexual disorders, defecatory disorders, anatomical restoration, and the quality of life of patients after surgery. This study aimed to evaluate the manometry results of patients before and after reconstructive surgery of the posterior compartment.

## MATERIAL AND METHODS

### Study population

This retrospective cohort study included women with defecation disorders referred to the Imam Khomeini Hospital Complex, affiliated with Tehran University of Medical Sciences, Tehran, Iran, from 2020 to 2021. The inclusion criteria were: 1) patients undergoing posterior compartment surgery; 2) willingness to participate in the study; 3) willingness to perform manometry; 4) absence of systemic diseases causing constipation or defecatory disorders; 5) at least stage II rectocele during the examination and 6) the presence of functional constipation symptoms. Functional constipation is often characterized by difficulty or infrequent bowel movements, pain during fecal passage, and/or feeling as though stools are not completely cleared. Patients unwilling to undergo manometry and those with systemic and chronic diseases were excluded from the study.

### Study protocol

The level II posterior defect was treated by repairing the rectovaginal fascia and perineal body, and level III DeLancey was connected to the uterosacral ligament or level I DeLancey. Variables assessed before and after surgery included incomplete stool evacuation, straining during defecation, the need to apply finger pressure to the vaginal or pelvic floor for a full evacuation, pain during defecation, and frequency of bowel movements. Also, the amount of pelvic prolapse was determined along with clinical symptoms based on the pelvic organ prolapse quantification system (POP-Q). Follow-up data, including recovery rates and new findings, were documented one year post-operation. An annual manometry was also performed for patients who had undergone the surgery.

### Posterior colporrhaphy technique

Level II posterior defects were treated by repairing the rectovaginal fascia (through the vagina) and connecting it to the pericervical ring at its caudal end (DeLancey level III to I).

The vagina and uterus were examined under general anesthesia in the lithotomy position. Transvaginal hysterectomy was conducted as the initial step when necessary. In cases requiring concurrent perineorrhaphy, surgical clamps were positioned at the 4 and 8 o'clock positions along the highest point of the vaginal epithelium at the posterior midline. The placement of the clamps was adjusted to ensure that the vaginal caliber accommodated two fingers comfortably for accurate assessment. An injection of normal saline was administered for hydro dissection, and a longitudinal incision was made at the midline of the prolapse with a surgical blade so that the underlying fibromuscular tissue could be accessed. Next, fibromuscular tissue was separated from the vaginal epithelium using scissors. The vaginal epithelium was separated as thinly as possible. The incision was initiated perpendicularly to the vaginal epithelium and as close to the Allis clamp to achieve the appropriate dissection depth. The vaginal epithelium was separated from the rectovaginal fascia throughout the posterior vaginal wall. The separation of the posterior epithelium of the vagina from the rectovaginal fascia and rectum continued until it reached the pericervical ring and both uterosacral ligaments so that both sides of the uterosacral ligaments could be touched. In the presence of an enterocele, the sac was separated from the posterior epithelium of the vagina. If sacrospinous ligament suspension was required, a right lateral dissection up to the right ischial spine was performed. Moreover, #0 Vicryl thread was used to repair large transversal defects in the rectovaginal fascia.

Subsequently, the rectovaginal fascia was sutured caudally from DeLancey levels I to III, parallel to the junction of the uterosacral ligaments to the posterior cervical lip or posterior part of the vaginal cuff, using 0-polydioxanone. Also, the uterosacral ligaments were sutured on both sides using 0-polydioxanone. Subsequently, the rectocele and enterocele (if present) were repaired. The vaginal epithelium was then corrected, if necessary, and the procedure was completed by closing the epithelium using #0 Vicryl. The uterosacral ligament, rectovaginal fascia, and perineal body were connected by vicryl suture. Cystoscopy was performed following the repair to ensure ureteral patency. A vaginal pack and urinary catheter were removed 24h after surgery.

### Statistical analysis

The descriptive data were presented as mean, standard deviation, and percentage. The normality of the data was checked prior to data analysis using the Shapiro-Wilk Test. Quantitative continuous variables with normal distribution were measured using Student’s t-test, and quantitative variables with non-normal distribution were measured using the Wilcoxon test. All the analyses were performed using the Statistical Package for the Social Sciences (SPSS) software, version 26. A p-value less than 0.05 was considered statistically significant.

## RESULTS

A total of 40 women, with a mean age of 49.47±9.66 years (age range: 31-71), participated in this study. Table 1 displays the distribution of parity among participants. The most common parity was three pregnancies (32.5%), followed by one pregnancy (2.5%), two pregnancies (27.5%), four pregnancies (22.5%), five pregnancies (5%), six pregnancies (2.5%), seven pregnancies (5%), and eight pregnancies (2.5%).

The manometric results of patients before and after surgery are presented in Table 1. There were no significant differences observed before and after surgery concerning maximum squeeze pressure (MSP) (p=0.5), first balloon sensation (FBS) (p=0.07), fecal urgency (FU) (p=0.15), and maximum tolerable volume (MTV) (p=0.04). However, significant differences were observed before and after surgery in terms of the maximum resting pressure (MRP) and push test (p<0.001), indicating that these parameters improved postoperatively.

**Table 1:** Manometric results of patients before and after surgery

Test		Before surgery N (%)	After surgery N (%)	p-value
Maximum squeeze pressure (MSP)	Normal	31 (77.5)	35 (87.5)	0.5
	Decreased	5 (12.5)	5 (12.5)	
	Increased	4 (10)	-	
Maximum resting pressure (MRP)	Normal	16 (40)	31 (77.5)	0.003
	Decreased	24 (60)	9 (22.5)	
	Increased	-	-	
First balloon sensation (FBS)	Normal	20 (50)	28 (70)	0.07
	Decreased	2 (5)	2 (5)	
	Increased	18 (45)	10 (25)	
Fecal urgency (FU)	Normal	11 (27.5)	20 (50)	0.15
	Decreased	2 (5)	3 (7.5)	
	Increased	27 (67.5)	17 (42.5)	
Maximum tolerable volume (MTV)	Normal	26 (65)	31 (77)	0.04
	Decreased	8 (20)	2 (5)	
	Increased	6 (15)	7 (17.5)	
Recto-anal inhibitory reflex (RAIR)	Abnormal	0 (0)	0 (0)	-
	Normal	40 (100)	40 (100)	
Push test	Normal	20 (50)	31 (77.5)	0.004
	Decreased	19 (47.5)	8 (20)	
	Increased	1 (2.5)	1 (2.5)	
Cough test	Abnormal	0 (0)	0 (0)	-
	Normal	40 (100)	40 (100)	

The results show significant improvements in constipation, straining for defecation, finger support during defecation, feeling of incomplete defecation, dyspareunia, and husband’s sexual satisfaction (p<0.001) postoperatively. Conversely, there were no significant changes in gas incontinence (p>0.05) and sexual activity (p=1.0), indicating that the intervention had no effect on these parameters (Table 2).

**Table 2:** Subjective results of patients before and after surgery

Test		Before surgery N (%)	After surgery N (%)	p-value
Constipation	Yes	40 (100)	8 (20)	<0.001
	No	0 (0)	32 (80)	
Straining for defecation	Yes	40 (100)	8 (20)	<0.001
	No	0 (0)	32 (80)	
Feeling of incomplete defecation	Yes	37 (92.5)	7 (17.5)	<0.001
	No	3 (7.5)	33 (82.5)	
Incomplete discharge	Yes	31 (77.5)	1 (2.5)	<0.001
	No	9 (8.8)	39 (97.5)	
Gas incontinence	Yes	1 (2.5)	0 (0)	>0.05
	No	39 (97.5)	40 (100)	
Sexual activity	Active	29 (72.5)	30 (75)	1.0
	Inactive	11 (27.5)	10 (25)	
Dyspareunia	Yes	28 (70)	10 (25)	<0.001
	No	12 (30)	30 (75)	
Husband’s sexual satisfaction	Yes	19 (47.5)	6 (15)	<0.001
	No	21 (52.5)	34 (85)	

Preoperative examination revealed that all 40 patients had posterior compartment prolapse grade ≥2, which improved in all cases after surgery (p>0.0001).

## DISCUSSION

This study evaluated the manometric results in patients with defecation disorders. The posterior level II defect was repaired by repairing the rectovaginal fascia and connecting it to the pericervical ring. This study showed that manometric parameters such as MRP, push test, constipation, straining during defecation, finger support during defecation, feeling of incomplete defecation, dyspareunia, and husband’s sexual satisfaction significantly improved after surgery. However, no improvement in MSP, FBS, FU, MTV, gas incontinence, or sexual activity was observed in patients after surgery.

Rectoceles can be treated with transvaginal, transanal, and abdominal repair, as well as graft materials for anatomical and functional purposes. The success rate, rationale, and complications of each approach, including anatomical cure, impact of defecation, and sexual function, have not been precisely described. Therefore, it is necessary to identify the best surgical technique for native vaginal tissue [13]. According to Sohbati *et al*., rectocele may occur in some women with posterior vaginal prolapse at a median age of 50 years [14]. This age was reported to be 61.5 and 65.4 years in the studies conducted by Khatri *et al*. [15] and Tan *et al*. respectively [8]. Consistent with previous studies, the mean age of the participants in this study was 49.47 years.

Rectoceles can be repaired surgically in the posterior vaginal compartment in several ways. However, there is no consensus on the optimal surgical technique. Moreover, clinical practice must consider the short- and long-term functional effects of repair. Transvaginal rectocele repair via anterior levatorplasty led to no significant changes in MTV, MRP, and MSP six months after surgery [16]. According to a review article, a median symptom improvement rate of 72.7% was observed after modifications to classical repair, which included omitting levatorplasty, adding the implant, concurrently performing lateral internal sphincterotomy, changing the direction of the rectovaginal septum plication, and site-specific surgery [17]. Another study reported that 58.2% of patients had improved symptoms of obstructive defecation after surgery and that rectocele recovery on ultrasound imaging was associated with improved symptoms of defecation disorder. However, they did not observe a relationship between rectocele repair and obstructive disorders [18].

Manometry and POP-Q examinations three months after posterior vaginal prolapse surgery showed that patients without mesh recovered better in anorectal function. Although no significant difference was observed in the MSP and MRP before and after surgery, anal pressure during voluntary defecation improved significantly in patients undergoing vaginal prolapse surgery [19]. According to the study findings, pelvic floor dysfunction is caused by a variety of factors other than structural changes. Therefore, anorectal pressure during defecation may be a better indicator of the effect of posterior pelvic floor repair. Short-term follow-up examinations showed significant improvements both anatomically and in terms of prolapse symptoms [20]. Significant improvements in urinary incontinence were observed after site-specific repairs of levels I and II in patients who underwent posterior vaginal wall compartment surgery with or without repair of other vaginal compartments with prolapse. However, the inferior defects did not improve significantly after the repair [21].

Based on the results obtained in this study, the MRP, push test, constipation, straining during defecation, finger support during defecation, feeling of incomplete defecation, dyspareunia, and husband’s sexual satisfaction improved after surgery. Therefore, rectovaginal fascia repair and connection to the pericervical ring surgery improved these outcomes. This is consistent with a study by Sohbati *et al*., which reported significant improvements in scores of the Pelvic Floor Distress Inventory (PFDI-20) and various symptoms such as fecal obstruction, urinary frequency, urinary incontinence, and swelling following surgery in a similar patient population. In this study, a comparison of the quantitative results of the POP-Q assessment before and after surgery in the follow-up period showed similar improvements in patients’ conditions after surgery [14].

According to the results of other studies, sexual dysfunction and dissatisfaction are common symptoms of pelvic organ prolapse [22, 23]. Various studies have shown that surgery has varying effects on women’s sexual satisfaction [23, 24]. Van Dam *et al*. reported that 41% of patients who underwent combined transvaginal/transanal rectocele repair had dyspareunia after surgery [25]. In a study by Shahghaibi *et al*., a statistically significant difference was observed in all sexual symptoms before and after colporrhaphy surgery [26]. Regarding sexual concerns, which included dyspareunia, and the overall satisfaction of women engaging in sexual activity, the surgical intervention had positive outcomes, with no postoperative reports of dyspareunia.

In this study, women with rectoceles underwent rectovaginal fascia repair connected to the pericervical ring. To the best of our knowledge, this is the first time that the results of this surgical technique have been studied for more than 12 months. Some of the limitations of this study are the short-term follow-up and the small sample size, making it difficult to generalize the results obtained in this study. Therefore, to develop and plan more appropriate comprehensive solutions based on epidemiological information, we recommend that future studies with larger sample sizes and longer follow-up periods be conducted at clinics in more centers.

## CONCLUSION

Based on the results obtained during the short-term follow-up period, we found that the surgery used in this study was effective and resulted in no significant complications. Clinical examinations revealed that the surgery improved manometry results, including MRP, push test, constipation, straining during defecation, finger support during defecation, feeling of incomplete defecation, and dyspareunia before and after surgery. The results of this study not only indicated a positive effect of the surgical procedure on patients and highlighted improvements in various parameters. However, more extensive studies should be conducted on manometry as a diagnostic tool in patients with defecatory disorders and posterior prolapse. Moreover, manometry can be used as a diagnostic tool for defecation disorders.

## Personal thanks

We would like to thank all the people who participated in this study.
